# Supplementary material for: DAO-CP: Data-Adaptive Online CP decomposition for tensor stream
Source: PLoS One. 2022 Apr 14;17(4):e0267091. doi: 10.1371/journal.pone.0267091 (PMC9009670; doi:10.1371/journal.pone.0267091)
Supplement: Attachment — Submitted filename: review.pdf [file pone.0267091.s001.pdf]

Referee Report

DAO-CP: Data-Adaptive Online CP Decomposition for Tensor Stream

by S Son, Y Park, M Cho, and U Kang
Submitted to PLOS ONE

In this paper, the authors describe an adaptive approach for computing CP decompositions of streaming tensors which they call DAO-CP. The problem setup is similar to many other works, where it is assumed a new tensor of fixed dimension is observed every time step, and when these tensors are stacked along a mode (the first mode in this paper), only that mode grows in time (called the temporal mode). The authors employ the DTD method from the MAST paper for updating the CP decomposition at each time step, restricted to the case of single temporal mode. They then build in logic for adapting the CP factor matrices due to changes in the distribution of the tensor data over time (which they call themes). The paper includes a description of the overall algorithm, a study of the computational complexity, and several experiments comparing to DTD, OnlineCP, and full CP decompositions.

Overall the paper is fairly easy to follow, and is consistent in notation, terminology, and theme as many other works in this area. Since the streaming decomposition method is basically DTD, the primary technical contribution of the paper is the logic for adapting the decomposition based on error thresholds when the current factor matrices do not well represent the new tensor data. Their logic for the adaptation process is reasonable, but does include some hyperparameters that would require tuning for each new problem. I think that contribution is significant enough to merit publication, but I do have several comments.

1. The approach does not appear to be appropriate for infinite data streams because by my reading of Eq. 12, all prior time steps are included in first term defining the objective function for the optimization problem solved each time step. Typically, authors use an exponential down-weighting of older and older time steps to effectively truncate the prior temporal data that must be stored and manipulated. Furthermore, this can be seen in Lemma 2, showing a cost proportional to I_1^{old}, which would grow linearly in time. It seems to me this would be straightforward improvement, so I wonder why the authors chose not to do this.

2. It is also unusual that the authors update the factor matrix rows corresponding to the prior time steps (see first line after Eq. 12). Typically, authors use a 2-stage process where they update the temporal mode keeping the non-temporal mode fixed (which means the rows of the factor matrix corresponding to old time steps do not change), and then update the non-temporal modes using the newly updated temporal factor. This saves computational cost, so again, I wonder why the authors chose not to do this.

3. The authors should make it clear their approach does not adapt the rank of the CP decomposition, nor add or remove factors. They should discuss how their method compares to the concept drift approach in *SeekAndDestroy* (Parischa, Gujral, Papelaxakis, 2019).

4. The computational results compare to running the full CP decomposition each time step using the entire streamed tensor to that point, which isn't necessary. A more fair comparison would be just running full CP at the end of the stream, since running it at intermediate time steps does not provide any new information.
