# Supplementary material for: DAO-CP: Data-Adaptive Online CP decomposition for tensor stream
Source: PLoS One. 2022 Apr 14;17(4):e0267091. doi: 10.1371/journal.pone.0267091 (PMC9009670; doi:10.1371/journal.pone.0267091)
Supplement: Attachment — Submitted filename: rebuttal_letter.pdf [file pone.0267091.s002.pdf]

DAO-CP: Data-Adaptive Online CP Decomposition for Tensor Stream

Sangjun Son, Yong-chan Park, Minyong Cho, and U Kang

We would like to thank the reviewers for their high quality reviews and constructive comments. Below, we summarize how we addressed the reviewers' requirements.

1. Reviewer 1.
   - **(R1-1)** The format of this article is not standard. If you use word template, please align left and right.
     - **(A1-1)** We corrected the format of the article according to reviewer's comments.
   - **(R1-2)** The writting is bad. I am not so sure about your contributions after reading the paper. your contribution is just the rules to detect the check points with local error norm? You use alternating minimization for DAO-CP.
     - **(A1-2)** We revised the paper to clearly highlight the contributions of our work (lines 29-35). Our main contributions are summarized as follows: (1) We employ z-score analysis to rapidly detect the change points of streaming tensors. (2) We introduce re-decomposition process to balance the trade-off between accuracy and speed. (3) We use complementary matrices and simplify the objective function from DTD in order to reduce the redundant computations in CP-ALS optimization.
   - **(R1-3)** Due to your method is data adaptive, it is better to give an depict of the data, which may affect the computation of local error norm?
     - **(A1-3)** We further included description of the data in Experiments section (lines 308-326). For example, Fig. 6 illustrates how our proposed method adapts to the Sample Video dataset; DAO-CP achieves the state-of-the-art accuracy when an object starts moving or a scene changes in the data.
   - **(R1-4)** It may be better to add some comparisons with some existing online methods.
     - **(A1-4)** We included another recent paper called "Identifying and Alleviating Concept Drift in Streaming Tensor Decomposition (R. Pasricha et al., 2019)," and compared it with our proposed method in Related Works section (lines 116-125).

2. Reviewer 2.
   - **(R2-1)** The approach does not appear to be appropriate for infinite data streams because by my reading of Eq. 12, all prior time steps are included in first term defining the objective function for the optimization problem solved each time step. Typically, authors use an exponential down-weighting of older and older time steps to effectively truncate the prior temporal data that must be stored and manipulated. Furthermore, this can be seen in Lemma 2, showing a cost proportional to I_1^{old}, which would grow linearly in time. It seems to me this would be straightforward improvement, so I wonder why the authors chose not to do this.
   - **(R2-2)** It is also unusual that the authors update the factor matrix rows corresponding to the prior time steps (see first line after Eq. 12). Typically, authors use a 2-stage process where they update the temporal mode keeping the non-temporal mode fixed (which means the rows of the factor matrix corresponding to old time steps do not change), and then update the non-temporal modes using the newly updated temporal factor. This saves computational cost, so again, I wonder why the authors chose not to do this.
     - **(A2-1 and A2-2)** We choose to consider all prior time steps and update the whole temporal factors to further increase the accuracy of decomposition. If the previous temporal factors are not updated, they harm the accuracy of method whenever there is a change of theme because they are optimized only for the previous theme of the data. Thus, even though our proposed method requires a time cost growing linearly, it significantly increases the decomposition

accuracy (Fig. 4), and shows promising speeds comparable to state-of-the-art methods for all the datasets (Fig. 5). We included additional explanation to justify our method in Proposed Method section (lines 190-193).

- **(R2-3)** The authors should make it clear their approach does not adapt the rank of the CP decomposition, nor add or remove factors. They should discuss how their method compares to the concept drift approach in *SeekAndDestroy* (Parischa, Gujral, Papelaxakis, 2019).
  - **(A2-3)** Unlike SeekAndDestroy, DAO-CP does not require estimating the actual rank numbers, but only tracks the difference of them using local error norms. This technique enables DAO-CP to rapidly capture the change points of theme without adding or removing factors. We included additional comparisons between DAO-CP and *SeekAndDestroy* in Related Works section (lines 116-125).
- **(R2-4)** The computational results compare to running the full CP decomposition each time step using the entire streamed tensor to that point, which isn't necessary. A more fair comparison would be just running full CP at the end of the stream, since running it at intermediate time steps does not provide any new information.
  - **(A2-4)** We compared the computation results between DAO-CP and full CP for each time step because we aim to answer how well DAO-CP performs for time-evolving tensors. In Fig. 6, we find that DAO-CP outperforms full CP in terms of local error norm whenever refinement or split process is executed. If we run full CP only at the end of the stream, we cannot compare the local error norm between DAO-CP and full CP at intermediate time steps.
